# Understanding Health Workers’ Job Preferences to Improve Rural Retention in Timor-Leste: Findings from a Discrete Choice Experiment

**DOI:** 10.1371/journal.pone.0165940

**Published:** 2016-11-15

**Authors:** Marc-Francois Smitz, Sophie Witter, Christophe Lemiere, Patrick Hoang-Vu Eozenou, Tomas Lievens, Rashid U. Zaman, Kay Engelhardt, Xiaohui Hou

**Affiliations:** 1 Oxford Policy Management, Oxford, United Kingdom; 2 Queen Margaret University, Edinburgh, United Kingdom; 3 World Bank, Washington DC, United States of America; Universidade Federal do Rio de Janeiro, BRAZIL

## Abstract

**Background:**

Timor-Leste built its health workforce up from extremely low levels after its war of independence, with the assistance of Cuban training, but faces challenges as the first cohorts of doctors will shortly be freed from their contracts with government. Retaining doctors, nurses and midwives in remote areas requires a good understanding of health worker preferences.

**Methods:**

The article reports on a discrete choice experiment (DCE) carried out amongst 441 health workers, including 173 doctors, 150 nurses and 118 midwives. Qualitative methods were conducted during the design phase. The attributes which emerged were wages, skills upgrading/specialisation, location, working conditions, transportation and housing.

**Findings:**

One of the main findings of the study is the relative lack of importance of wages for doctors, which could be linked to high intrinsic motivation, perceptions of having an already highly paid job (relative to local conditions), and/or being in a relatively early stage of their career for most respondents. Professional development provides the highest satisfaction with jobs, followed by the working conditions. Doctors with less experience, males and the unmarried are more flexible about location. For nurses and midwives, skill upgrading emerged as the most cost effective method.

**Conclusions:**

The study is the first of its kind conducted in Timor-Leste. It provides policy-relevant information to balance financial and non-financial incentives for different cadres and profiles of staff. It also augments a thin literature on the preferences of working doctors (as opposed to medical students) in low and middle income countries and provides insights into the ability to instil motivation to work in rural areas, which may be influenced by rural recruitment and Cuban-style training, with its emphasis on community service.

## Introduction

Rural retention of health staff, and especially more highly trained health staff such as doctors, nurses and midwives, is a challenge in most low income countries and especially fragile and post-conflict states, which have often lost much of their trained health workforce [1, 2]. Comprehensive international guidelines to improve retention have been drawn up by the World Health Organisation [3]. However, there is recognition that health labour markets are very varied [4], and that it is important to discern local preferences in order to plan human resources for health policies [5]. This paper reports on the preferences of doctors, nurses and midwives in the context of Timor-Leste. The objective is to provide policy makers with knowledge about health workforce preferences in order to take cost-effective measures to retain medical staff in remote areas. We report here on the results of a discrete choice experiment (DCE), which was embedded in a wider health labour market study. While the use of DCEs to study health worker preferences is growing [6], this is the first study of the health workforce of Timor Leste, which may exhibit interesting characteristics.

Timor-Leste became independent in 2002 and faced the challenge of a collapsed health system with only a handful of doctors [7]. During the war preceding independence more than 70% of the country’s health facilities were seriously damaged and only approximately 20 doctors remained in the country [8].

This radical shortage of health workers gradually started to improve when Cuban medical doctors arrived in the country as part of an international aid program. In 2010, the health worker density in Timor-Leste was 1.3 per 1,000 population [9], which was below the WHO minimum recommended threshold of 2.3 [10]. In the neighbouring country Indonesia, the density in 2010 was 2.11 per thousand people [11]. AAs the first waves of Timorese trained in Cuba by the Cuban Medical Brigade (CMB) started to return to Timor-Leste in 2010, the number of health workers has significantly increased: in 2014, the health worker density was 2.02 per thousand people. However, the density varied considerably by district–from 0.04 doctors per thousand people to 0.35, while for nurses (0.4–1.4) and midwives (0.2–0.9) the distribution was slightly more even [12]. While the initial massive shortage has now been minimised, there are concerns over more complex issues including facility functionality, rural retention, skills imbalance, motivation, preferences and competence of the health workers. A survey was therefore carried out recently in order to understand the labour market dynamics among health workers, to learn more about their preferences and concerns, and to assess the skills, competence and motivation of doctors. This article reports on the discrete choice component of the survey, which is a standard technique used to elicit preferences for job attributes.

The health sector in Timor-Leste is divided into four organizational levels: district health services, hospitals, Central services, and personalized services [12]. District health services are primarily responsible for delivering the basic health service package. They operate 66 community health centres (CHCs), 192 health posts, mobile clinics as well as the *Sistema Integradu Saude Communitaria (SISCa)*. SISCa, introduced in late 2007, is a community based programme that delivers preventative and curative health services. The distribution of health posts around the country is designed so that every citizen has a health facility within walking distance. Sparsely populated areas are served by mobile clinics and SISCas. The district health services work with the Central services in implementing a wide range of programs such as child immunization, malaria and TB programmes.

After the independence conflict, the government of Timor-Leste sought an international partnership to help rebuild its health care system. As part of its international development program, Cuba proposed to train around a thousand medical doctors at the *Escuela LatinoAmericana de Medicina* (ELAM) in Cuba and also in the *Universidade Nasionál Timór Lorosa* (UNTL) in Timor-Leste [12,13]. The selection of the students started with an application to enter the programme: any young high school graduate in the country was eligible. 677 doctors were enrolled between 2004 and 2007 for training in Cuba and 328 for Cuban training in Timor-Leste between 2005 and 2011 [9], Admission to the program was not based on an exam, but the place of origin, as per the letter of recommendation of village authorities. It is recognised that rural origin is a strong determinant of work place selection for medical staff [14]. Finally, medical students signed a contract, which was a *sine qua non* condition for participation. The contract stipulated that medical doctors have to work for at least six years for the government [15]. The training provided was focused on community health and social medicine, i.e. not only curative health, but also public health, especially preventive medicine. In addition to theoretical knowledge, a sense of social duty was developed during the course of their studies [16, 17]. On graduation, a large majority were assigned to Health Posts (HP) and Community Health Centres (CHC) in rural areas, often to the area they came from. They received a motorbike, salaries and sporadic visits from more senior doctors, especially from Cuba.

The government has set itself the objective of providing free universal coverage to all the Timorese by 2020. It plans to have a medical doctor, two nurses and two midwives in each Health Post of the country [18]. In 2014, the mean density of doctors, nurses and midwives in the HPs were 1.1, 0.7 and 0.8 respectively. In this context, the Ministry of Health (MoH) is facing the challenge of the increasing financial burden of a growing health workforce, alongside the need to retain the early cohorts, which will soon be freed from their contract to work in the public sector. MoH also has to tackle the lack of motivation of health workers located in remote areas, where cases of absenteeism are reported (personal communication, MoH officials).

In these circumstances, it is crucial for the Ministry of Health to align incentives to retain medical staff in the remote areas in the most efficient way possible. The main objective of this study was to elicit Timorese health workers’ preferences for job attributes and inform policy makers of Timor-Leste.The Timor-Leste results are also of wider interest as they can shed light on the preferences of workers trained purposefully to develop high motivation to work in preventive health and rural areas in the ethos of the Cuban Medical Brigades and deepen the literature on health worker preferences in low income and fragile contexts.

## Methods

### Design

A Discrete Choice Experiment (DCE) is an experiment in which respondents have to make various choices, each one between two (or more) options. The options have different levels on key characteristics (attributes). The aim is to reveal preferences and trade-offs between different factors which have value for respondents, and which are amenable to policy interventions.

To understand the context and define appropriate attributes for the DCE, qualitative work was first carried out in Timor-Leste. It entailed three focus group discussions (FGDs) with medical doctors, and two with nurses and one with midwives, in both rural and urban facilities. The FGDs participants were balanced in gender (except for midwives), workplace (health posts and community health centres) and age (except for MDs that were all under 30). Semi structured key informant interviews were conducted with officials from the MoH at national and district level, final year medical students and with medical doctors. All the interviews and FGDs were recorded and analysed following structured grids, gathering information on valuation, feasibility and scaling of job attributes.

The seven DCE attributes selected for doctors and nurses/midwives are summed up in Table 1. FGDs were conducted separately for nurses and midwives, but as their views were alike for the most part, we decided to amalgamate the two groups. However, doctors and nurses/midwives did have significant dissimilarities and it was appropriate to have different levels for some attributes. The health workers were open regarding all aspects of their job, such as the lack of supplies or housing. They were however more reluctant to talk about the wage, and they prefer to use terms such as “fair” wage. MDs seem to consider their current wage (610 per month) as fair, while nurses and midwives opinionated that their current wage (450 per month) was too low, relative to what young and inexperienced MDs earn. In the absence of any precise amount from health workers, the wage component of the DCE was based mostly on what the ministry of health could envision and afford: a 20% or a 40% increase from the current level.

**Table 1:** Attributes with the levels and description.

N	Attribute	N	*Levels*		Description
**1**	Location	1	*Urban*		urban places–including suburbs (Dili and Bacau)
		2	*Remote*		rural place that are accessible by motor vehicle all year long
		3	*Extremely remote*		rural places that are not accessible all year long with motor vehicles
**2**	Facility type	4	*CHC*		Based in a Community Health Center
		5	*HP*		Based in a Health Post
**3**	Health Facility Equipment	6	*Good level*		the wall and roof are in good condition, there is a water and electricity supply. The lab testing is separated from the main consultation office. Medical staff is provided with all standard test kits and drugs to diagnose and treat common diseases. They also have the instruments they need to diagnose patients (sphygmo, stethoscope, light, glucometer) and emergency response tools (oxygen, nebuliser, defibrillator), as well as medical clothes and masks
		7	*Medium level*		the physical condition of the building is not good, but medical staff are provided with all standard test kits and drugs to diagnose and treat common diseases. They also have the instruments they need to diagnose patients (sphygmo, stethoscope, light, glucometer) and emergency response tools (oxygen, nebuliser, defibrillator)
		8	*Poor*		the physical condition of the building is not good. Medical staff faces from time to time shortage of drugs, and often doesn't have tests to diagnose diseases. They have a few tools (stethoscopes, lights, needles,), but lack emergency response tools most of the time.
**4**	Housing	9	*Good housing*		individual room for each health worker (with one more room for the children as needed), a shared kitchen/common room. House's wall and roof in good shape. A door that closes and can be locked.
		10	*Poor housing*		health workers share same rooms, wall and roof are not in good condition, entrance door is not secured enough
**5**	Transportation	11	*Motorbike*		MoH provides a motorbike
		12	*No motorbike*		MoH does not provides a motorbike
** **			***MD***	***Nurses / Midwives***	
**6**	income	13	*610 USD*	*450 USD*	wage per month
		14	*732 USD*	*540 USD*	wage per month
		15	*854 USD*	*630 USD*	wage per month
**7**	Training	16	*none*		no training, low probability of specializing
		17	*Workshops*		Every year, attend a few of workshops on specific themes (EMOC, Emergency for MD, complicated deliveries for midwives)
		18	*visits from specialist*	* *	Weekly visit from specialists in you CHC/HP to discuss and address difficult cases encountered
		19	*higher edu*	*bachelor degree*	For MD: Higher probability than the other doctors to be selected for specialisation. For nurses/midwives: finish bachelor degree (if uncompleted)

One important lesson of the qualitative work is that health workers had difficulties to decontextualize their choice from their current situation. In order to tackle this issue, a job similar to the average job was used as a constant comparator (job A), and only the other jobs (jobs B) varied in their attribute levels. Using a constant comparator is frequent in the DCE literature. [19].

Health workers were asked to make 16 binary choices, as most studies using single blocking use between 16 and 20 choice tasks [6]. This number is sufficient to estimate the 12 main effects of the experiment.

In order to extract the most of each observation, it is required to have the lowest variance possible in the attribute/levels matrix, given the expected coefficient [20, 21]. The design optimisation was performed using the macros %mkt* [21] for SAS, which maximise the D-efficiency. A constraint was imposed to make sure that attributes “health centre” and “extremely remote area” never appear together. Finally, the wage component was modified in a one of two set in order to improve utility balance, which increases the information obtain at a very low D-efficiency cost [22]. Finally, after making a choice, an option “would refuse both jobs” was also proposed to respondents, which can be used to test robustness of the results. The choice sets, as well as an example of a choice presented to doctors, can be found in S1 Table.

### Tools

The DCE module was part of a larger health worker survey in Timor-Leste that involved a facility level questionnaire, a health worker questionnaire and a Direct Clinical Observation. The data collection tools went through extensive pretesting (to finalize the design of the instruments), piloting (to check the applicability in the field) and field practice (for the fieldworkers) before they were implemented. The survey took place in July and August 2014. The DCE was the first module of the survey in order to avoid any bias that may occur from positioning of the respondent [23]. The interviews were carried out individually maintaining privacy and confidentiality of the responses that were provided. Interviews were conducted in Tetun. The respondents were given two cards (namely Job A and Job B) in each set with the attributes written in it. They were asked to choose one of the jobs.

### Sampling

The sample was drawn from six strata: two kinds of facilities (urban and rural) and three kinds of health workers (doctors, midwives, nurses). We obtained the sampling frame from the Ministry of Health; it included 2,247 health workers in the country, of which 612 were doctors, 1,095 nurses and 540 midwives. The health workers were randomly sampled from 69 health facilities in all 13 districts of Timor-Leste using Probability Proportionate to Size (PPS). Replacement occurred within the same facility if the sampled worker could not be interviewed. The final sample included 441 health workers, including 173 doctors, 150 nurses and 118 midwives.

### Analysis

ur model assumes that individuals (n = 1…N) face a choice between the two different jobs (i and j). The Random Utility Theory [24] stipulates that they will choose the option that provides them with the highest utility, i.e. individual n will pick option *i* if U_ni_ > U_nj._. The choice made is based on observable characteristics of each option (vectors Xi and Xj), as well as unobserved parameters Ɛ_i_ and Ɛ_j_.

$$U_{i}=\alpha +{\beta X}_{i}+\varepsilon_{i}\;and\;U_{j}=\alpha +{\beta X}_{j}+\varepsilon_{j}$$ (1)

The probability to pick job i over job j therefore relies on both the observable and unobservable parameters:

$${Prob}_{n\;i/j}=Prob(U_{ni}>U_{nj})=Prob(\beta (X_{ni}-X_{nj)})>\varepsilon_{nj}-\varepsilon_{ni}=F(\beta (X_{ni}-X_{nj)})$$ (2)

We assume that unobserved term Ɛ_nj_−Ɛ_ni_ follows the cumulative density function (F) of the logistic density function. We estimated this equation with a maximum likelihood using Stata12 software. We used a conditional logit modelling [25] to take into account the grouping of each set. We addressed within respondent correlation in responses by clustering at the individual level. The econometric modelling of DCE does not allow the inclusion of any control, as each choice made by each respondent is the highest independent level of observation. We used interaction terms between the attributes and specific subsample of the population to investigate heterogeneity of preferences.

The Marginal Rate of Substitution (MRS) between two attributes is given by the ratio of their coefficient.

$${MRS}_{X1/X2}=-\frac{{\beta^{\prime }}_{2}}{{\beta^{\prime }}_{1}}$$ (3)

As for the probability to select job i over job j, it is derived from the logistic function and takes the following form [26].

$$P_{ij}=\frac{e^{\left(\beta X_{i}\right)}}{\sum_{j}e^{\left(\beta X_{j}\right)}}$$ (4)

### Ethics

The study protocol was approved by Oxford Policy Management’s Ethical Review Committee and by the Human Research Ethics Committee of the Institute of Health of Timor-Leste. All the participants provided informed written consent prior to the interviews.

## Results

### Descriptive statistics

More than 9 in 10 doctors interviewed were trained by the Cuban Medical Brigades, and 44% have an urban background. Doctors are relatively young (29 years) and 63% are married. Nurses and midwives are on average 11 years older than the doctors and only a third of them come from Dili, the capital (Table 2).

**Table 2:** Demographic charecteristics of the health workers participating in the DCE.

Health workers type:	MDs		Nurses		Midwives		total	
	Mean	SD	Mean	SD	Mean	SD	Mean	SD
female	58.4%	0.49	30.7%	0.46	100.0%	0.00	60.1%	0.49
age	28.9	3.82	41.4	9.90	37.6	7.73	35.5	9.20
married	63.6%	0.48	86.0%	0.35	84.7%	0.36	76.9%	0.42
Parents from urban area	43.9%	0.50	30.0%	0.46	32.2%	0.47	36.1%	0.48
Trained by Cuban Medical Brigades	92.5%	0.26	1.3%	0.12	0.0%	0.00	36.7%	0.48
Health faciltiy: Health Post	15.6%	0.36	12.0%	0.33	15.3%	0.36	14.3%	0.35
Health facility: Community Health Center	41.0%	0.49	66.7%	0.47	53.4%	0.50	53.1%	0.50
Health facility: Hospital	43.4%	0.50	21.3%	0.41	31.4%	0.47	32.7%	0.47
Health Facility in Rural Area	39.9%	0.49	52.7%	0.50	44.9%	0.50	45.6%	0.50
Lack of drugs	84.4%	0.36	82.7%	0.38	80.5%	0.40	82.8%	0.38
Heavy workload	17.3%	0.38	16.0%	0.37	12.7%	0.33	15.6%	0.36
Has housing	32.4%	0.47	7.3%	0.26	19.5%	0.40	20.4%	0.40
Has good housing	20.2%	0.40	5.3%	0.23	8.5%	0.28	12.0%	0.33
Professional Motorbike	43.4%	0.50	27.3%	0.45	7.6%	0.27	28.3%	0.45
N	173		150		118		441	

An average of 39% of the medical doctors would choose the constant job (A): the minimum is 23.7% for set number 3 and the maximum is around 56.6% for set 11. The nurses and midwives had averages of 54.5% and 53.1% respectively, and their ranges were 42%–65% and 46%–64% respectively. The job selection rates can be found in S2 Table. The fact that health workers were found to be split over the choices indicates that there was some balance in utility between the jobs proposed: some trade-offs were made by health workers and the data should carry a lot of information.

For each set, health workers were also asked whether they would refuse both jobs. On average, 16.5% of the set proposed would be rejected by health workers. Rejection rate is higher for midwives (20%) than for nurses (13%), which might reflect that their current status is slightly better. The variation of refusal rate per set is small, ranging from -5% to +5% around the health worker type mean, so any difference in the robustness analysis will not be driven by a higher drop off rate of some sets.

### Model estimation

#### Regression results for doctors

The results from the regression show that the most valued items are related to training and education. They would prefer training through a higher probability to enter a specialisation program, or benefit from the visit of specialists, but not being delivered information through workshops. This preference for training is driven mainly by female, single and urban doctors (Table 3). In terms of work location, doctors would prefer it to be urban, especially females of urban origin. As for the workplace, good equipment is strongly valued by female doctors and doctors who practice in rural facilities, probably due to poorer conditions there. Community Health Centres are also significantly preferred to health posts. Regarding the direct personal incentives for doctors, it is surprising to find that good housing is the only item that emerges as statistically significant. Motorbikes are not an attractive attribute. Wage is overall non-significant, but there too, a significant gender gap in the preferences is observed: while woman have a statistically significant distaste for money (p<0.01), their male counterpart found it to be the most attractive attribute, as it is to them the only positively significant attribute.

**Table 3:** Regression, MRS and robustness check.

**Regressions: MDs**														
	**All**			**would accept**	**Interaction: Gender**		**Interaction: Marital status**		**Interaction: 2+ years experience**		**Interaction: Urban family**		**Interaction: Rural facility**	
	**coef**	**MRS**	**MRS p value**	**coef**	**base (58%)**	**i.male**	**base (36%)**	**i.married**	**base (61%)**	**i.2Y exp**	**base (56%)**	**i.urban family**	**base (60%)**	**i.rural facility**
Remote location	0.087	-0.330	0.944	0.102	0.105	-0.052	-0.090	0.291	0.136	-0.177	0.170	-0.199	-0.008	0.173
Urban location	0.265**			0.281*	0.277	-0.028	0.144	0.199	0.252	0.041	0.351**	-0.202	0.205	0.112
Medium equipment	0.304***	-1.149	0.410	0.372***	0.481***	-0.419*	0.374*	-0.111	0.224	0.289	0.207	0.230	0.477***	-0.312
Good equipment	0.374***	-1.413	0.277	0.428***	0.552***	-0.424*	0.179	0.322	0.305**	0.245	0.293*	0.188	0.507***	-0.243
Facility: CHC	0.227**	-0.858	0.591	0.285***	0.110	0.285	0.273	-0.071	0.199	0.101	0.261*	-0.080	0.352***	-0.225
Workshops	0.180	-0.680	0.747	0.220*	0.217	-0.077	0.126	0.089	0.193	-0.048	0.215	-0.074	0.046	0.249
Visits from specialists	0.523***	-1.978	0.173	0.573***	0.633***	-0.252	0.479**	0.074	0.491***	0.115	0.484**	0.098	0.437***	0.161
Specialization	0.763***	-2.883	0.080	0.866***	0.858***	-0.220	0.636***	0.212	0.898***	-0.477	0.749***	0.037	0.580***	0.341
Good housing	0.317***	-1.200	0.378	0.330***	0.427***	-0.254	0.172	0.237	0.264**	0.199	0.278**	0.095	0.412***	-0.169
Motorbike	0.026	-0.098	1.000	0.027	0.026	-0.001	-0.107	0.214	0.058	-0.112	0.052	-0.063	-0.018	0.084
Total wage	-0.000			-0.000	-0.001***	0.003***	-0.000	-0.000	-0.000	0.000	0.000	-0.001*	-0.001	0.001
N	2,768			2,290	2,768		2,768		2,768		2,768		2,768	
**Regressions: Nurses & Midwives**														
	**All**			**robust**	**Gender**		**marital status**		**Experience**		**Urban family**		**Rural facility**	
	**coef**	**MRS**	**MRS p value**	**coef**	**base (61%)**	**i.male**	**base (15%)**	**i.married**	**base (19%)**	**i.5Y exp**	**base (69%)**	**i.urban family**	**base (51%)**	**i.rural facility**
Remote location	0.086	-$73.8	0.464	0.119	0.047	0.089	-0.162	0.295	0.473*	-0.486*	-0.034	0.404*	0.017	0.201
Urban location	0.066	-$56.2	0.559	0.056	0.028	0.083	-0.159	0.268	0.512**	-0.559**	-0.060	0.419*	0.064	0.006
Medium equipment	0.075	-$64.0	0.343	0.113	0.110	-0.078	-0.127	0.240	-0.230	0.382*	0.117	-0.141	-0.032	0.308**
Good equipment	-0.027	$22.7	0.763	0.018	-0.045	0.042	-0.006	-0.024	-0.136	0.137	0.066	-0.308**	-0.081	0.159
Workshops	0.195**	-$166.5	0.215	0.273***	0.264***	-0.154	0.225	-0.035	0.184	0.015	0.237**	-0.139	0.192*	0.011
Bachelor's degree	0.067	-$57.4	0.416	0.077	0.075	-0.018	-0.097	0.194	-0.122	0.238	0.005	0.208	-0.032	0.288*
Facility: CHC	0.131*	-$112.3	0.278	0.152*	0.180*	-0.108	0.124	0.009	-0.120	0.316	0.212**	-0.267	0.094	0.110
Good housing	0.136**	-$116.3	0.080	0.122*	0.103	0.074	0.150	-0.015	0.128	0.010	0.149*	-0.045	0.108	0.079
Motorbike	0.105	-$89.8	0.103	0.091	-0.004	0.243*	-0.105	0.249	-0.260*	0.457***	0.153*	-0.158	0.151*	-0.134
Total wage	0.001			0.001	0.001	0.001	-0.000	0.002	-0.002*	0.004***	0.002**	-0.003*	0.001	0.002
N	4,288			3,596	4,288		4,288		4,288		4,288		4,288	

note: pval< .01 ***; pval < .05 **; pval < .1 *;

The Marginal Rates of Substitution describe the exchange rate of an attribute for the other. It is usual in the literature to use the monetary component as the benchmark comparator which serves to price the other job attributes. We cannot proceed as such here because the wage component has an unusual low value and is not different from zero. Splitting the analysis by gender doesn’t make sense either, as wage has a negative coefficient for woman, and it is the only positive coefficient for man. We decided to report the substitution rates for the urban location component. Several attributes are more valuable than an urban assignment: but only specialisation would significantly (p<0.1) compensate for a non-urban location.

#### Regression results for nurses/midwives

The nurses and midwives have more usual preferences, as they value the wage attribute positively (although not statistically significantly), which enables the monetary valuation of the other attributes. Additional training through workshops ($167) is the most valued item, while obtaining a bachelor degree is less important ($57), as many health workers have graduated already and might not feel concerned by this attribute. Good housing ($136 wage equivalent) and a motorbike ($90) are also valued positively. Facility type also carries some importance, as working in CHC is valued at $112 per month. They prefer to work either in urban or remote areas ($56 and $73, respectively), but this is significant only for nurses and midwives from urban background. Equipment comes last.

#### Simulations

Using formula (4) above, we simulated the natural allocation of doctors, nurses and midwives in the different livelihood zones, assuming that all other attributes are held constant. We found that 29% of the medical doctors would choose to be in an extremely remote area, 32% in a remote area and 38% in an urban area. The small differences between the probabilities indicate that the concern about urban-rural imbalance that motivated this study is not explained by the location *per se*, but rather by the fact that living and working conditions are different in urban, remote and very remote facilities.

To investigate this, we released the *ceteris paribus* assumption and let the other attributes be more realistic by creating a “base scenario”, i.e. jobs that best reflect the current actual conditions associated with each livelihood zone (Table 4). The extremely remote job has low level of equipment and little support, the remote job is slightly better: located in a community health centre with a medium level of equipment, doctors benefit from good housing and visits from specialists and a medium level of equipment, while nurses and midwives attend workshops. The urban job, although located in a health post, has good level of equipment and higher training opportunities. The natural equilibrium under these circumstances is that only 9.4% of the doctors would join the extremely remote job, and 50% would stay in urban areas. For nurses and midwives, the imbalance would be less severe, but only 25% of them would work in extremely remote areas.

**Table 4:** Base typical jobs.

Base typical jobs						
	Medical Doctors			Nurses and Midwoves		
Location	ext remote	remote	urban	ext remote	remote	urban
Equipment	poor	medium	good	poor	medium	good
training	none	visits	Visits + workshops	none	workshops	workshops
housing	poor	good	good	None	None	None
facility	HP	CHC	HP	HP	CHC	HP
moto	Y	Y	Y	No	No	No
wage	610	610	610	450	450	450

This major imbalance indicate that there could be some relocation of doctors away from extremely remote areas, as doctors are progressively released from the contract that binds them to the government. We simulated rural retention policies to find ways to avoid this imbalance. We decided to keep the remote and urban jobs constant and improve the extremely remote job. The new equilibriums were computed using the formula (4) described above.

The first scenario invests in better infrastructure such as equipment and housing: it would increase health workers’ prevalence in extremely remote areas to reach 16% for doctors and 30% for nurses and midwives. The second scenario entails specialists’ support and a higher probability of specialisation for doctors. The effect is dramatic: 27.5% of the doctors would purposely go to extremely remote areas to benefit from the educational opportunities. Providing workshops to extremely remote area nurses and midwives would convince an additional 7% of them to reallocate there. The mix-scenario for doctors would add medium level equipment to the higher education scenario. It would balance the human resources in the country, with about one third in each livelihood zone. The same balance could be reached for nurses and midwives by increasing personal compensations—a 20% increase in wages, a motorbike and good housing—or by providing both infrastructure improvement and educational opportunities (Table 5).

**Table 5:** Simulated improved jobs in extremely remote health posts.

Simulated improved jobs in extremely remote health posts						
Scenarios description	Medical Doctors			Nurses		
	Infrastructure	Human capital	Mixed	Infrastructure	Human capital	personal benefits
Location	ext remote	ext remote	ext remote	ext remote	ext remote	ext remote
Equipment	medium	poor	medium	medium	poor	poor
Training	none	Visits + specialisation	Visits + specialisation	none	Workshops + bach degree	none
Housing	good	poor	poor	good	poor	good
Facility	HP	HP	HP	HP	HP	HP
Motorbike	Y	Y	Y	No	No	Yes
Wage	610	610	610	450	450	540
**Allocation**	ext remote	Remote	Urban	ext remote	Remote	Urban
benchmark	9.5%	40.7%	49.8%	25.7%	41.8%	32.5%
Infrastructure sc.	16.3%	37.7%	46.0%	29.9%	39.4%	30.6%
Human capital sc.	27.5%	32.6%	39.9%	31.0%	38.8%	30.2%
Mixed sc.	33.9%	29.7%	36.3%	32.8%	37.8%	29.4%

S3 Table reports the allocation of different subgroups, for the baseline scenario as well as for the three scenarios described above. The imbalance between zones is more pronounced for female doctors, as only 7% of them would work in extremely remote areas under current circumstances. Improved infrastructure scenario would reduce significantly the gender gap, which is consistent with the unsafe housing that female doctors reported fearing during focus group discussion.

Across all scenarios, non-Cuban Medical Brigade trained doctors preferred working in remote area, and would even prefer extremely remote areas rather than urban areas. Cuban trained doctors preferred urban areas and are highly responsive to the human capital improvement scenario (i.e. the second scenario).

Health workers with more than two years of experience and those who are married are less likely to take an extremely remote position. Surprisingly, health workers with a parent in an urban area do not have a larger bias against extremely remote areas than their counterparts with rural origins. Job satisfaction is the single most relevant item: only 4% of the less satisfied doctors would go to extremely remote area under baseline scenario, while 18% of the satisfied group would be willing to do so.

## Discussion

This study investigated the preferences of health workers in Timor-Leste. It is innovative insofar as it is the only study of this type in this country. It is also the first DCE focusing on Cuban medical brigade trained doctors. Findings can also be linked to the wider results of the survey in terms of health worker characteristics and views, facility functionality and doctors’ competence and practices [27].

The survey reached its planned sample as 441 health workers completed the DCE module. The design also reached its objective to collect the preferences of health workers as health workers were split in their responses to each set of choice. The percentage of respondents who picked either only jobs A or only jobs B is very small (1.3%) and results are robust to their exclusion. Another indication that health workers made trade-offs is that very few picked jobs only according to one attribute’s level, except for the specialisation item of doctors which is indeed the highest valued item. Only 4% of health workers systematically picked the job with urban location, so the attribute of interest for this study was highly traded.

One limitation is that the qualitative fieldwork indicated that respondents found it hard to set themselves in an abstract situation, and tended to nest in their current working conditions: for example, they already have a motorcycle and tend to think they would retain it, even if the job attribute says there is no transportation. This could bias our results.

One of the most interesting findings of the study is the *relative* lack of importance of wages for doctors, especially female. Although most studies in the literature found the opposite, these results here are in line with two recent DCEs [1, 28]. However, this study adds to the literature insofar as the *absolute* importance of wages for practicing female doctors is null. This could be interpreted as high intrinsic (or non-financial) motivation or an already highly paid job. For instance, in the Timor-Leste context, doctors may see themselves as already better-off (with a salary of US$ 610 per month) compared to other civil servants. In that case, their preference for professional development opportunities rather than increased wages may be reasonable. Moreover, most of the doctors in Timor-Leste are in an early stage at their career–a stage when they may value an investment in their career development more than short term financial incentives. The role of the Cuban Medical Brigade training in developing intrinsic motivation cannot be identified clearly in this study. The fact that our sample contains only 12 non Cuban trained doctors who are working mainly for NGOs or religious organisation does not provide us with a sufficient sample to disentangle the Cuban training effect. However, we can note that Timorese nurses and midwives have more standard preferences and infer that the low valuation of wage is not a Timor-specific characteristic. Wage increases offered within the DCE to doctors were significant and large by local standards (20 and 40% of salary). In the FGD discussion doctors also indicated that they were happy with their current income and how it positions them in the socio-economic ladder. In FGDs, income also came last, so these results are coherent.

Professional development, either through skills acquired by senior doctors or by formal specialisation degree, provides the highest job satisfaction, followed by the working conditions. It provides important insights at a time when the MoH is at a crossroad and has to make choices related to its budget. The low level of financial motivation displayed by doctors may allow the MoH to focus on improving material conditions for health facilities instead of allocating resources to increase the wages of the current health work force.

The Ministry of Health is also currently exploring international partnership to train specialists in various medical fields. It should be aware that the selection of medical doctors for the programme is a very effective way to incentivise doctors to work in extremely remote areas. Transparency concerning the probability of being enrolled in specialisation is a key element for this policy to be effective.

Policies regarding nurses and midwives are less urgent as their labour market is not about to liberalise and the MoH has no reason to fear mass reallocation. However, improving their satisfaction and optimising incentives is always important. Skill upgrading through workshops may be a cost effective method to act on the nurses’ and midwives’ labour market, as it is both highly valued and relatively cheap to set up. By comparison, a DCE amongst nurses in Liberia found pay and transportation as the key elements, along with selecting candidates of rural origin [29].

The differences between different sub-samples also generate useful knowledge. Doctors with less experience, males and the unmarried are more flexible about location. Labour market planning can use this information, while still ensuring that experienced medical doctors are equitably distributed across geographical areas in order to ensure equitable access to health care services. Although the design does not allow robust estimation of joint attributes, FGDs clearly highlighted the importance of housing and transportation for extremely remote areas.

## Conclusion

The Ministry of Health can use the information generated by this study to find an appropriate balance between the financial and non-financial benefits that might influence health workers to work in rural areas and stay motivated. The DCE results indicate that for nurses, midwives and medical doctors, skill upgrading either through formal studies or through workshops is likely to be an effective strategy. Improving the facilities’ equipment and drug availability would not only improve the health situation of the country, but also strongly improve job satisfaction of health workers, as well as make jobs in remote locations more attractive. The results are also of interest as the wage attribute for doctors is not significantly different from zero, which is uncommon in the DCE literature. We can only speculate that it is linked to the background, the training received and/or the current salary level of the doctors in Timor Leste, which appear to meet the expectation of the young doctors.

## Supporting Information

S1 File Data files (Stata 13). (ZIP)

S1 Table Choice sets table and an example. (DOCX)

S2 Table Job selection rate table, by choice set and HW type. (DOCX)

S3 Table Allocation of Health Workers’ subgroups under different scenarios. (DOCX)
