# Supplementary material for: Understanding Health Workers’ Job Preferences to Improve Rural Retention in Timor-Leste: Findings from a Discrete Choice Experiment
Source: PLoS One. 2016 Nov 15;11(11):e0165940. doi: 10.1371/journal.pone.0165940 (PMC5112867; doi:10.1371/journal.pone.0165940)
Supplement: S1 Table — (DOCX) [file pone.0165940.s002.docx]

**S1: Choice sets**

| **Choice set for MDs** | | | | | | | |
| --- | --- | --- | --- | --- | --- | --- | --- |
| **set #** | **Location** | **HF type** | **Transport** | **Housing** | **Equipment** | **Wage** | **Training** |
| 1 | Urban | CHC | moto | good | medium | $854 | none |
| 2 | Remote | CHC | none | good | poor | $732 | visit from specialists |
| 3 | Urban | CHC | none | poor | medium | $610 | specialisation |
| 4 | Ext. remote | HP | moto | poor | good | $854 | specialisation |
| 5 | Urban | CHC | none | poor | poor | $854 | workshops |
| 6 | Ext. remote | HP | moto | good | poor | $854 | specialisation |
| 7 | Urban | HP | moto | poor | poor | $610 | none |
| 8 | Urban | HP | none | good | good | $732 | specialisation |
| 9 | Urban | HP | moto | poor | good | $854 | visit from specialists |
| 10 | Urban | CHC | moto | poor | poor | $732 | visit from specialists |
| 11 | Ext. remote | HP | none | poor | good | $732 | none |
| 12 | Remote | CHC | moto | good | good | $610 | workshops |
| 13 | Urban | HP | moto | good | poor | $610 | none |
| 14 | Remote | HP | none | good | medium | $854 | visit from specialists |
| 15 | Ext. remote | HP | moto | poor | medium | $610 | visit from specialists |
| 16 | Ext. remote | HP | none | good | good | $610 | workshops |
|  |  |  |  |  |  |  |  |
| constant | Remote | HP | moto | poor | medium | $732 | workshops |
|  |  |  |  |  |  |  |  |
| **Choice set for Nurses and Midwives** | | | | | | | |
| **set #** | **Location** | **HF type** | **Transport** | **Housing** | **Equipment** | **Wage** | **Training** |
| 1 | remote | HP | moto | good | good | $450 | none |
| 2 | urban | CHC | none | poor | medium | $630 | none |
| 3 | remote | HP | none | poor | poor | $630 | none |
| 4 | urban | HP | moto | good | medium | $450 | Bachelor degree |
| 5 | urban | HP | none | good | good | $545 | Bachelor degree |
| 6 | Ext. remote | HP | none | good | good | $630 | workshop |
| 7 | Ext. remote | HP | moto | poor | good | $450 | Bachelor degree |
| 8 | remote | CHC | none | good | medium | $450 | none |
| 9 | Ext. remote | HP | none | poor | medium | $450 | Bachelor degree |
| 10 | remote | CHC | none | good | poor | $545 | Bachelor degree |
| 11 | urban | HP | none | poor | poor | $630 | workshop |
| 12 | remote | CHC | moto | poor | poor | $630 | Bachelor degree |
| 13 | remote | CHC | none | good | good | $630 | workshop |
| 14 | urban | CHC | moto | good | poor | $450 | workshop |
| 15 | urban | CHC | moto | poor | good | $545 | none |
| 16 | Ext. remote | HP | moto | good | poor | $545 | none |
|  |  |  |  |  |  |  |  |
| constant | Remote | HP | moto | poor | medium | $545 | workshops |
|  |  |  |  |  |  |  |  |
|  |  |  |  |  |  |  |  |
| **Numeru 1 / 16** | | | | | | | |
| **Posizaun 1** | | | | **Posizaun 2** | | | |
| Fatin | | **Rural** | | Fatin | | **Vila** | |
| Servisu iha | | **Postu Saude** | | Servisu iha | | **Sentru Saude Komunitariu** | |
| Transporte | | **Motor** | | Transporte | | **Motor** | |
| Uma | | **La diak** | | Uma | | **Diak** | |
| Ekipamentu saude | | **Diak uitoan** | | Ekipamentu saude | | **Diak uitoan** | |
| Salariu | | **$732** | | Salariu | | **$854** | |
| Simu formasaun | | **Workshop** | | Simu formasaun | | **La simu** | |
