# Supplementary material for: Understanding Health Workers’ Job Preferences to Improve Rural Retention in Timor-Leste: Findings from a Discrete Choice Experiment
Source: PLoS One. 2016 Nov 15;11(11):e0165940. doi: 10.1371/journal.pone.0165940 (PMC5112867; doi:10.1371/journal.pone.0165940)
Supplement: S2 Table — (DOCX) [file pone.0165940.s003.docx]

**S2: Job selection rate, by choice set and HW type**

|  | | | | | | |
| --- | --- | --- | --- | --- | --- | --- |
|  | **Doctor** | | **Nurses** | | **Midwives** | |
| **set** | **choice** | **refuse** | **choice** | **refuse** | **choice** | **refuse** |
|  |  |  |  |  |  |  |
| 1 | 37.6% | 13.9% | 62.7% | 10.7% | 63.6% | 16.9% |
| 2 | 42.2% | 16.8% | 55.3% | 7.3% | 46.6% | 16.1% |
| 3 | 23.7% | 16.2% | 57.3% | 9.3% | 58.5% | 19.5% |
| 4 | 34.1% | 20.2% | 54.0% | 12.7% | 60.2% | 23.7% |
| 5 | 46.8% | 20.8% | 48.7% | 13.3% | 52.5% | 22.0% |
| 6 | 37.6% | 23.1% | 54.0% | 10.0% | 55.9% | 20.3% |
| 7 | 51.4% | 20.2% | 64.0% | 15.3% | 59.3% | 19.5% |
| 8 | 31.2% | 17.3% | 56.0% | 16.0% | 51.7% | 16.9% |
| 9 | 35.8% | 13.3% | 64.7% | 14.0% | 50.8% | 22.9% |
| 10 | 41.6% | 15.0% | 45.3% | 16.0% | 50.8% | 22.9% |
| 11 | 56.6% | 17.3% | 58.0% | 10.0% | 50.8% | 22.0% |
| 12 | 26.6% | 16.2% | 52.7% | 17.3% | 48.3% | 25.4% |
| 13 | 48.0% | 14.5% | 42.0% | 13.3% | 45.8% | 18.6% |
| 14 | 26.0% | 20.8% | 52.7% | 15.3% | 49.2% | 13.6% |
| 15 | 42.2% | 15.6% | 50.7% | 14.7% | 53.4% | 22.9% |
| 16 | 42.8% | 15.0% | 54.7% | 12.8% | 51.7% | 17.8% |
|  |  |  |  |  |  |  |
| Total | 39.0% | 17.3% | 54.5% | 13.0% | 53.1% | 20.1% |
