# Supplementary material for: Understanding Health Workers’ Job Preferences to Improve Rural Retention in Timor-Leste: Findings from a Discrete Choice Experiment
Source: PLoS One. 2016 Nov 15;11(11):e0165940. doi: 10.1371/journal.pone.0165940 (PMC5112867; doi:10.1371/journal.pone.0165940)
Supplement: S3 Table — (DOCX) [file pone.0165940.s004.docx]

**S3: Allocation of Health Workers’ subgroups under different scenarios**

| **MEDICAL DOCTORS** | | | | | | | |
| --- | --- | --- | --- | --- | --- | --- | --- |
|  | % health workers | **Base scenario** | | | **Infrastructure scenario** | | |
|  |  | ext remote | remote | urban | ext remote | remote | urban |
| All | 100% | 9% | 41% | 50% | 16% | 38% | 46% |
| Male | 42% | 15% | 42% | 43% | 18% | 41% | 41% |
| Female | 58% | 7% | 39% | 55% | 15% | 35% | 50% |
| Cuban Med Brig | 92% | 9% | 40% | 51% | 16% | 37% | 47% |
| Non Cuban Med Brig | 8% | 18% | 71% | 10% | 30% | 61% | 9% |
| Less than 2 years exp | 62% | 11% | 40% | 49% | 17% | 38% | 46% |
| More than 2 years exp | 38% | 7% | 42% | 52% | 16% | 38% | 47% |
| Parents in Urban | 44% | 9% | 41% | 50% | 18% | 37% | 45% |
| Parents in Rural | 56% | 10% | 40% | 50% | 15% | 38% | 47% |
| HW married | 64% | 7% | 37% | 55% | 13% | 35% | 52% |
| HW unmarried | 36% | 14% | 46% | 41% | 21% | 41% | 37% |
| Urban facility | 60% | 9% | 47% | 44% | 19% | 42% | 39% |
| Rural facility | 40% | 10% | 36% | 54% | 14% | 34% | 52% |
| Never worked in HP | 92% | 10% | 41% | 49% | 16% | 38% | 45% |
| Worked in HP | 8% | 6% | 34% | 60% | 17% | 30% | 53% |
| Low job satisfaction | 50% | 4% | 34% | 62% | 10% | 32% | 59% |
| High job satisfaction | 50% | 18% | 44% | 38% | 24% | 41% | 35% |
| No housing provided | 68% | 9% | 37% | 53% | 18% | 34% | 48% |
| Housing provided | 32% | 9% | 46% | 45% | 14% | 44% | 42% |
| Good housing provided | 20% | 6% | 46% | 49% | 11% | 43% | 46% |
|  |  |  |  |  |  |  |  |
|  | % health workers | **Human capital scenario** | | | **Mixed scenario** | | |
|  |  | ext remote | remote | urban | ext remote | remote | urban |
| All | 100% | 27% | 33% | 40% | 34% | 30% | 36% |
| Male | 42% | 32% | 34% | 34% | 34% | 33% | 33% |
| Female | 58% | 24% | 31% | 45% | 34% | 27% | 39% |
| Cuban Med Brig | 92% | 28% | 32% | 41% | 34% | 29% | 37% |
| Non Cubab Med Brig | 8% | 16% | 73% | 11% | 25% | 65% | 10% |
| Less than 2 years exp | 62% | 33% | 30% | 37% | 38% | 28% | 34% |
| More than 2 years exp | 38% | 17% | 37% | 46% | 25% | 34% | 42% |
| Parents in Urban | 44% | 28% | 33% | 39% | 37% | 29% | 34% |
| Parents in Rural | 56% | 27% | 32% | 40% | 32% | 30% | 38% |
| HW married | 64% | 24% | 30% | 45% | 29% | 28% | 42% |
| HW unmarried | 36% | 32% | 36% | 32% | 41% | 31% | 28% |
| Urban facility | 60% | 21% | 41% | 38% | 30% | 36% | 34% |
| Rural facility | 40% | 33% | 27% | 40% | 37% | 25% | 38% |
| Never worked in HP | 92% | 28% | 33% | 39% | 34% | 30% | 36% |
| Worked in HP | 8% | 20% | 29% | 51% | 34% | 24% | 42% |
| Low job satisfaction | 50% | 22% | 27% | 51% | 31% | 24% | 45% |
| High job satisfaction | 50% | 33% | 36% | 31% | 36% | 35% | 30% |
| No housing provided | 68% | 26% | 31% | 43% | 33% | 28% | 39% |
| Housing provided | 32% | 30% | 36% | 34% | 36% | 33% | 31% |
| Good housing provided | 20% | 33% | 32% | 35% | 43% | 27% | 29% |
|  |  |  |  |  |  |  |  |
| **Nurses and midwives** | | | | | | | |
|  | % health workers | **Base scenario** | | | **Infrastructure scenario** | | |
|  |  | ext remote | remote | urban | ext remote | remote | urban |
| All | 100% | 26% | 42% | 32% | 32% | 36% | 32% |
| Male | 39% | 27% | 39% | 34% | 28% | 42% | 30% |
| Female | 61% | 24% | 44% | 31% | 32% | 23% | 45% |
| Less than 2 years exp | 7% | 23% | 27% | 51% | 30% | 41% | 30% |
| More than 2 years exp | 93% | 26% | 43% | 31% | 29% | 39% | 33% |
| Parents in Urban | 31% | 27% | 40% | 33% | 30% | 40% | 30% |
| Parents in Rural | 69% | 25% | 43% | 32% | 29% | 41% | 30% |
| HW married | 85% | 25% | 43% | 32% | 33% | 34% | 34% |
| HW unmarried | 15% | 32% | 34% | 34% | 30% | 37% | 33% |
| Urban facility | 51% | 29% | 37% | 34% | 29% | 45% | 26% |
| Rural facility | 49% | 20% | 50% | 29% | 32% | 38% | 31% |
| Never worked in HP | 68% | 29% | 39% | 32% | 26% | 43% | 30% |
| Worked in HP | 32% | 19% | 48% | 33% | 29% | 42% | 29% |
| Low job satisfaction | 46% | 22% | 46% | 32% | 31% | 37% | 32% |
| High job satisfaction | 54% | 30% | 38% | 33% | 29% | 39% | 32% |
| No housing provided | 87% | 25% | 41% | 34% | 34% | 45% | 21% |
| Housing provided | 13% | 27% | 50% | 23% | 35% | 37% | 27% |
| Good housing provided | 7% | 26% | 43% | 31% | 29% | 39% | 32% |
|  |  |  |  |  |  |  |  |
|  | % health workers | **Human capital scenario** | | | **Mixed scenario** | | |
|  |  | ext remote | remote | urban | ext remote | remote | urban |
| All | 100% | 31% | 39% | 30% | 33% | 38% | 29% |
| Male | 39% | 31% | 37% | 32% | 40% | 32% | 28% |
| Female | 61% | 31% | 40% | 28% | 28% | 42% | 30% |
| Less than 2 years exp | 7% | 13% | 30% | 57% | 23% | 26% | 50% |
| More than 2 years exp | 93% | 33% | 39% | 28% | 33% | 39% | 28% |
| Parents in Urban | 31% | 33% | 36% | 30% | 28% | 39% | 33% |
| Parents in Rural | 69% | 30% | 40% | 30% | 35% | 37% | 28% |
| HW married | 85% | 30% | 40% | 30% | 33% | 39% | 29% |
| HW unmarried | 15% | 35% | 33% | 33% | 32% | 34% | 34% |
| Urban facility | 51% | 32% | 36% | 32% | 35% | 34% | 31% |
| Rural facility | 49% | 29% | 45% | 26% | 28% | 46% | 26% |
| Never worked in HP | 68% | 34% | 36% | 29% | 34% | 36% | 29% |
| Worked in HP | 32% | 24% | 45% | 31% | 30% | 41% | 29% |
| Low job satisfaction | 46% | 30% | 41% | 29% | 33% | 39% | 28% |
| High job satisfaction | 54% | 32% | 36% | 31% | 33% | 36% | 31% |
| No housing provided | 87% | 31% | 38% | 31% | 33% | 37% | 30% |
| Housing provided | 13% | 30% | 48% | 22% | 27% | 50% | 23% |
| Good housing provided | 7% | 33% | 39% | 29% | 29% | 41% | 30% |
